# *Chlamydia psittaci* and *C. avium* in feral pigeon (*Columba livia domestica*) droppings in two cities in the Netherlands

**DOI:** 10.1080/01652176.2018.1482028

**Published:** 2018-06-05

**Authors:** Sara A. Burt, Romy E. Röring, Marloes Heijne

**Affiliations:** a Division of Environmental Epidemiology & Veterinary Public Health, Institute for Risk Assessment Sciences, Faculty of Veterinary Medicine, Utrecht University, Utrecht, The Netherlands;; b Department of Bacteriology and Epidemiology, Wageningen Bioveterinary Research, Lelystad, The Netherlands

**Keywords:** Pigeon, feral, *Chlamydia psittaci*, *Chlamydia avium*, psittacosis

## Abstract

**Background:** Feral pigeons (*Columba livia domestica*) live and breed in many city centres and contact with their droppings can be a hazard for human health if the birds carry *Chlamydia psittaci.*

**Objective:** The aim of this study was to establish whether pigeon droppings in two Dutch cities (Utrecht and Haarlem) contain *C. psittaci* and/or *C. avium*, which could be a potential hazard for transmission to humans.

**Methods:** In May 2017 seven feral pigeon ‘hot spots’ with between 5 and 40+ pigeons present were identified in two cities by visual observations over two days. During the following ten days fresh droppings were collected at these hot spots and the samples were pooled per three droppings to achieve 40–41 samples per city. Samples were analysed for *Chlamydia* DNA with a broad range 23S *Chlamydiaceae* Real-Time PCR and positive samples were tested with a specific *C. psittaci* and *C. avium* Real-Time PCR. Positive *C. psittaci* samples were genotyped.

**Results:***C. psittaci* and *C. avium* were detected in both cities. For *C. psittaci* the prevalences in Utrecht and Haarlem were 2.4% and 7.5%, respectively; for *C. avium* 36.6% and 20.0%, respectively. One sample contained both species. All *C. psittaci* samples belonged to genotype B.

**Conclusion:***C. psittaci* and *C. avium* are present in feral pigeon droppings in Utrecht and Haarlem. Human contact with droppings from infected pigeons or inhalation of dust from dried droppings represent a potential hazard to public health.

## 1. Introduction

Many people enjoy seeing and feeding the feral pigeons (*Columba livia domestica*) that live in city centres and parks. However, contact with pigeons or their droppings can be a health hazard since the birds can carry zoonoses such as microsporidia (Bart et al. 2008), *Campylobacter* spp. (Dudzic et al. 2016), *Salmonella* spp. (Haesendonck et al. 2016), and *Chlamydia psittaci* (Heddema et al. 2006a). *Chlamydia* spp. are Gram-negative obligate intracellular bacteria (Harkinezhad et al. 2009). *Chlamydia psittaci* is the causative organism of psittacosis (ornithosis) and *C. avium* is a recently identified species for which the zoonotic potential has not yet been identified (Sachse et al. 2014). The number of human cases of psittacosis in the Netherlands over the last ten years has varied between 40–80 per year (Rijksinstituut voor Volksgezondheid en Milieu 2017) but *C. psittaci* may be underdiagnosed as a cause of community-acquired pneumonia because testing for psittacosis is often not included in the routine diagnosis (Landelijke Coordinatiestructuur Infectieziektebestrijding 2007; van der Hoek et al. 2014; Spoorenberg et al. 2016).

*C. psittaci* is subdivided into at least nine different genotypes based on sequencing of the outer membrane protein A (*OmpA*) gene (Sachse et al. 2008; van Lent et al. 2012). Each genotype is associated with different hosts, for example genotype A is associated with parrots, genotype B with pigeons and some wild birds and genotype C with ducks (Radomski et al. 2016). The present genotyping system only accounts for part of the genetic diversity of *C. psittaci*. An extension has been proposed, which would include subgroups to genotypes A, E/B and D, and six new (provisional) genotypes to include strains that are presently not typable (Sachse et al. 2008).

Avian associated genotypes of *C. psittaci* can cause infection in humans and it has been suggested that non-psittacine birds are an underestimated source of infection (Heddema et al. 2006b; Radomski et al. 2016). The contribution of poultry farms to human infection has not yet been fully clarified (Hogerwerf et al. 2017; Heijne et al. 2018) and there is some evidence that contact with garden birds may contribute to zoonotic transmission (Rehn et al. 2013). Of the Dutch psittacosis patients recorded between 2008–2016, approximately 6% reported contact with wild pigeons (Heddema et al. 2015; Rijksinstituut voor Volksgezondheid en Milieu 2017). In 15–25 cases each year the animal source of infection is unknown or not reported (Rijksinstituut voor Volksgezondheid en Milieu 2017); it is possible that some of these patients contracted psittacosis from pigeons via the urban environment.

The last survey of the prevalence of *C. psittaci* in feral pigeons in urban areas in the Netherlands was carried out in Amsterdam more than 10 years ago (Heddema et al. 2006a). Then, the prevalence in a total of 331 feral pigeon droppings collected at nine locations in the city averaged 5% (95% CI, 2–10%) during the late winter and 10% (95% CI, 6–16%) during the spring breeding season.

The aim of this study was to establish whether pigeon droppings in two Dutch cities (Utrecht and Haarlem) contain *C. psittaci* and/or *C. avium*, which could be a potential hazard for transmission to humans.

## 2. Materials and methods

In May 2017, feral pigeon ‘hot spots’ with at least five and up to more than 40 pigeons present were identified in two Dutch cities, Utrecht and Haarlem, by visual observations over two days based on the method of Buijs and van Wijnen (Buijs and van Wijnen 2001). At most of these locations food was sold, cafes were situated or the location was a park in which people fed pigeons. In one case it concerned a viaduct where the birds nested. During the succeeding 10 days fresh droppings were collected at seven hot spots in each city and frozen at −20 °C until analysis. The samples were pooled per three droppings resulting in 41 pooled samples from Utrecht and 40 pooled samples from Haarlem.

DNA isolation was performed as described earlier (Heijne et al. 2018) and tested for the presence of *Chlamydia* DNA with a broad range 23S *Chlamydiaceae* Real-Time PCR (Ehricht et al. 2006). Subsequently, positive samples were tested with a specific *C. psittaci* and *C. avium* Real-Time PCR (Pantchev et al. 2009; Zocevic et al. 2013). *C. psittaci* positive samples were genotyped according to the method of Heddema et al. (Heddema et al. 2015).

## 3. Statistical analysis

The difference between the frequencies of positive samples in the two cities were tested for significance using the two sample Z-test on the Epitools website (http://epitools.ausvet.com.au/content.php?page=home). A p-value below 0.05 was considered significant.

## 4. Results

The results of the PCR analysis for *C. psittaci* and *C. avium* are presented in Table 1. Overall, *C. psittaci* was detected in 4/81 (4.9%) and *C. avium* in 23/81 (28.4%) pooled samples of feral pigeon droppings. One sample from Haarlem contained both species. All *C. psittaci* samples belonged to genotype B.

**Table 1.** Prevalences of *Chlamydia psittaci* and *C. avium* in pooled samples of feral pigeon droppings from two cities in the Netherlands.

	*C. psittaci*			*C. avium*		
	No. of samples positive	Percentage positive	95% CI	No. of samples positive	Percentage positive	95% CI
Utrecht	1/41	2.4%^a^	0–7.2%	15/41	36.6%^b,c^	21.8–51.3%
Haarlem	3/40	7.5%^a^^,c^	0–15.7%	8/40	20.0%^c^	7.6–32.4%

^a,b,c^Percentage prevalences indicated by a different letter are significantly different (p < 0.05).

The difference in the frequencies of positive pooled samples between the two cities was not significant for either species (p > 0.05). In Utrecht, *C. avium* was found significantly more often than *C. psittaci* (p < 0.0001), whereas in Haarlem the difference in prevalence between the two species was not significant (p > 0.05).

## 5. Discussion

In this study several pooled samples of pigeon droppings from the centre of two cities were found positive for *C. psittaci* genotype B and a much larger proportion of the samples was positive for *C. avium.*

The *C. psittaci* prevalence we found in Haarlem is in accordance with the range of prevalences found in pigeons in Amsterdam, namely between 5% (in February) and 10% (in May) (Heddema et al. 2006a). However, it should be noted that the present study used pooled samples from three pigeons, whereas the Amsterdam study sampled individual pigeon droppings. Prevalences from the city of Moers in Germany were found to be much higher: 46.7%–76.7% (Sachse et al. 2012). Our sampling was carried out during May, when most birds are breeding. At other times of year excretion of *Chlamydiae* may be lower because it is known that stress during the breeding period can stimulate excretion of *Chlamydiae* (European Commission 2002).

*C. psittaci* genotype B found here is quite usual for pigeons (Vanrompay et al. 1993). The dried droppings from feral pigeons in these two cities represent a potential source of infection with *C. psittaci* if they are inhaled (Harkinezhad et al. 2009). Although *Chlamydia* spp. are obligate intracellular bacteria, *C. psittaci* can survive long enough outside the body to make transmission via the environment possible (Harkinezhad et al. 2009; Szymanska-Czerwinska and Niemczuk 2016). Much is yet unclear about the uptake of *C. psittaci* by host cells and it has not yet been established how many *Chlamydiae* must be inhaled to become infected (Radomski et al. 2016).

This appears to be the first report of *C. avium* in feral pigeons in the Netherlands but the species has been identified retrospectively in pigeon samples in Germany between 1996–2012 (Sachse et al. 2014). *C. avium* has only recently been identified as a separate species and the potential for contributing to human chlamydiosis is unknown (Sachse et al. 2014).

More research is needed to determine whether action should be taken to reduce the chance of human contact with pigeon droppings and/or to treat feral pigeons to reduce carriage of *C. psittaci*. A more thorough diagnosis of human pneumonia cases would enable better evaluation of the source of infection and provide more information on the relative contribution of feral pigeons to human psittacosis. Sampling individual birds would provide a more accurate assessment of the *C. psittaci* prevalence in the city pigeon populations. It would also be interesting to evaluate whether the use of pigeon lofts in city centres to attract pigeons away from the areas where most people are (market squares and parks), has an effect on the prevalence in the birds or on the spreading of *Chlamydia* spp. in the environment. For example, environmental dust collectors could be used around these areas to investigate the potential spread of these bacteria in the environment, which is a likely route of transmission to humans.

## Conclusion

*C. psittaci* and *C. avium* are present in droppings of feral pigeons in two Dutch cities. Human contact with contaminated pigeon droppings, either directly or by inhalation of dust from dried droppings, represents a potential hazard for public health.

## Data Availability

The data that support the findings of this study are available from the corresponding author, SB, upon reasonable request.
